# A Multi-Methodological MR Resting State Network Analysis to Assess the Changes in Brain Physiology of Children with ADHD

**DOI:** 10.1371/journal.pone.0099119

**Published:** 2014-06-19

**Authors:** Benito de Celis Alonso, Silvia Hidalgo Tobón, Pilar Dies Suarez, Julio García Flores, Benito de Celis Carrillo, Eduardo Barragán Pérez

**Affiliations:** 1 Faculty of Physics and Mathematics, Benemérita Universidad Autónoma de Puebla (BUAP), Puebla, Mexico; 2 Imaging Department, Hospital Infantil de México Federico Gómez, México DF, Mexico; 3 Feinberg School of Medicine, Northwestern University, Chicago, Illinois, United States of America; 4 School of Industrial Engineering, Universidad de León, León, Castilla y León, Spain; 5 Neurology Department, Hospital Infantil de México Federico Gómez, México DF, Mexico; Hospital General Dr. Manuel Gea González, Mexico

## Abstract

The purpose of this work was to highlight the neurological differences between the MR resting state networks of a group of children with ADHD (pre-treatment) and an age-matched healthy group. Results were obtained using different image analysis techniques. A sample of n = 46 children with ages between 6 and 12 years were included in this study (23 per cohort). Resting state image analysis was performed using ReHo, ALFF and ICA techniques. ReHo and ICA represent connectivity analyses calculated with different mathematical approaches. ALFF represents an indirect measurement of brain activity. The ReHo and ICA analyses suggested differences between the two groups, while the ALFF analysis did not. The ReHo and ALFF analyses presented differences with respect to the results previously reported in the literature. ICA analysis showed that the same resting state networks that appear in healthy volunteers of adult age were obtained for both groups. In contrast, these networks were not identical when comparing the healthy and ADHD groups. These differences affected areas for all the networks except the Right Memory Function network. All techniques employed in this study were used to monitor different cerebral regions which participate in the phenomenological characterization of ADHD patients when compared to healthy controls. Results from our three analyses indicated that the cerebellum and mid-frontal lobe bilaterally for ReHo, the executive function regions in ICA, and the precuneus, cuneus and the clacarine fissure for ALFF, were the “hubs” in which the main *inter-group* differences were found. These results do not just help to explain the physiology underlying the disorder but open the door to future uses of these methodologies to monitor and evaluate patients with ADHD.

## Introduction

Attention deficit hyperactivity disorder (ADHD) is a common neuropsychiatric disorder in children and adolescents with a prevalence of 5.29% according to current meta-analysis studies [1]. Three main groups of symptomatologies have been found: Inattentiveness, Impulsivity-Hyperactivity and Combined. These symptoms are believed to change with age, driven by changes in brain structures and connectivity [2]. Unfortunately, an objective diagnostic tool useful to monitor this kind of patient is still lacking. It is therefore important to understand from a neurobiological angle the behavior of brain regions and their connections in these patients, allowing for information-based therapies. Brain resting state networks represent correlated slow fluctuations of the BOLD MR signal in different brain regions (frequencies between 0.01 and 0.1 Hz) [3], [4]. They are characterized by comprising regions involved in similar tasks, even when distant in the brain (i.e. vision, motor cortex, etc.). They are very plastic [5] and change with age [6]; [7]. These networks are consistently found in healthy volunteers [8], but they are known to be affected by drug abuse [9] and almost every kind of neurodegenerative disorder [10]; [11].

There are several analytical approaches (i.e. ReHo, ALFF, fALFF, independent component analysis, AFNI, etc.), as well as computing platforms (i.e. FSL, SPM, RETROICOR, etc.) that can be used to study resting state networks. In this study three of these approaches were used: firstly, Regions of Homogeneity (ReHo). In this approach the BOLD time course of each voxel of the brain (excluding CSF and white matter) is filtered, retaining frequencies between 0.01 and 0.1 Hz. Kendall correlation coefficients (a non-parametric test for correlation) for each voxel are then calculated with respect to its closest 27 neighbors. These neighbors represent a cube in 3D around the given voxel [12]. The result of the p-value for this correlation is then plotted on top of an anatomical image. Unlike in independent component analysis, in which Bayesian statistics are used to find covariance trends between voxels, ReHo analysis is a “simple” correlation calculation which will show all resting state networks together in the brain under study. The connectivity showed by a region and calculated with this technique will be limited to its neighbor regions in the brain. ReHo analysis is a widely known method and has been used, for example, in connectivity studies of deaf patients [13], epilepsy [14] and sensorimotor cortex [15]. The second approach used was ALFF (amplitude of low frequency fluctuations). The ALFF measurement is an indirect biomarker of brain activity. It shows not a correlation between regions as with ReHo, but rather the amplitude of the BOLD contrast in a given area. It has been found that ALFF activations were larger in the right paracentral lobule when volunteers lay in the scanner with eyes closed, compared to open-eyed volunteers [16]. Mathematically it is obtained by calculating the power spectra of the low frequency BOLD fluctuations. ALFF is a technique which has started to become available for neuroscientists, and has been used in studies of schizophrenia [17], vision [18] and neurological disorders like apathy or depression [19]. Thirdly, independent component analysis (ICA) is a computational and statistical method used to separate linearly mixed signals without an a priori model. When used on fMRI data, there are two approaches that separate the signals based on time or space variances. The second is the most common and has been successfully used in multiple studies: some examples to date include Wolf et al. [20] on ADHD adult patients, and studies of schizophrenia [21]. A review of the technique was compiled by Robinson et al. [22]. One of the early limitations of this technique was that it worked well on individual subjects but did not allow for data grouping. Calhoun et al. [23] developed algorithms that included data compression, ICA and back-reconstruction steps, which now make it possible to extend these analyses to group studies.

Several reviews [24]; [25]; [26] of resting state networks in ADHD patients indicate that several brain regions are more active or inactive when compared to healthy groups: larger ReHo was found in ADHD patients, compared to healthy volunteers, bilaterally in the dorsal anterior cingulate cortex, cerebellum, thalamus, pons and insula [27]. Other cases of larger homogeneities were found in the occipital cortex [28] and sensory and sensory-related cortexes [29]. ALFF was found to be increased in young ADHD patients in the right anterior cingulate cortex, the left sensorimotor cortex and bilaterally in the brainstem [30]. In contrast, healthy volunteers presented ReHo correlations, which did not appear in the ADHD group, in the frontal striatal cerebellum [28]. ICA analysis has become to be a useful tool to differentiate ADHD patients from healthy controls, and therefore is becoming more of a diagnostic tool [31]. Nevertheless, work with pediatric patients using ICA is sparse. A study on children with ADHD that used different imaging techniques (voxel based morphometry, diffusion tensor imaging and magnetization transfer; [32] has found four fMRI components from their ICA analysis. These components were divided into two groups: positively correlated with a working memory paradigm, which included the parietal and frontal regions; and negatively correlated, connecting anterior and posterior cingulate cortex with the precuneus, as well as temporal regions. The latter were thought to be part of the Default network. Another study on adult patients [20] has found a functional network comprising lateral prefrontal (bilaterally), striatal, and cingulate regions. ADHD adults, when compared with healthy controls, had lower connectivity bilaterally for the VLPFC region, the superior parietal lobe, the anterior cingulate cortex and the cerebellum. Increased connectivity in ADHD adults was found in the left dorsal cingulate cortex, the right prefrontal regions and the left cuneus. Recently, the ADHD 200 global competition (http://fcon_1000.projects.nitrc.org/indi/adhd200/) has made available free resting state data sets of 747 volunteers (healthy and ADHD with ages between 7 and 16 years old). This project aims at differentially diagnosing ADHD patients (with their corresponding subtype) from healthy controls. It has been able to identify the illness with a maximum efficiency till now of 69.59% [33]. It has also helped showing the implication in ADHD of structures in the ventral default network region (precuneus, para-hippocampal, and posterior cingulate) ([34]) ([35]).

One of the pre-existing hypotheses for ADHD is that a malfunction or subnormal performance of the dopaminergic and norepinephrinergic systems is the main source of the disorder. These problems affect primarily the prefrontal cortex which is responsible, amongst other things, for: inhibition, concentration, executive functions, motivation, memory, organizing, planning and problem solving [36]. The dopaminergic system has an important role in the control of motion, arousal, motivation and reward, through its activity in the substantia nigra in the basal ganglia (motion), and the ventral tegmental area (VTA) in the limbic system (motion, motivation and decision making; [37]. The norepinephrinergic system, based in the lateral tegmental area as well as the locus coeruleus, reaches almost all of the CNS [38]. It controls the reward system, arousal and motivation.

Work on ADHD has been predominantly performed in young adults/adolescents (ages 10–17; see [24], for a complete review), but not on children specifically. In this study we compared medication-naïve children with ADHD (ages between 6 and 12 years) to age and sex matched healthy ones. We analysed the inter-group differences in MR resting states with different analytical approaches (ReHo, ALFF and ICA). We highlighted some of the physiological differences of the ADHD brain when compared to a healthy one.

## Methods

### Volunteers

All experiments were performed according to the international conventions for biomedical studies on humans. The ethical research committee of the Hospital Infantil de México, Federico Gómez (México DF, México) granted the necessary ethical approval for this study under the title “Técnicas avanzadas de Resonancia Magnética Funcional en población pediátrica con diagnóstico de TDAH” (“Advanced functional MR techniques applied on patients diagnosed with ADHD”). This permission was obtained in June 2012, six months prior to the start of experiments which were performed over the first 6 months of 2013. When enrolling a volunteer to this study, an information session was first held, in which the task was explained to the volunteer and their legal guardian, and they were told what to expect from the experimental protocol, as well as its dangers. Secondly, they were asked to read at home the information sheets which repeated all of the information provided in the oral session. The language level of the oral sessions as well as the written information sheet was intended to be accessible for non-scientific or medically trained people. Finally, consent forms were signed by the legal guardians and handed to scientists in charge of the study. Twenty-seven (n = 27) ADHD patients together with 24 healthy volunteers (H from now on) were included in this study. One ADHD volunteer was excluded from the study as he could not remain in the scanner for the whole length of the protocol. Another four volunteers (three in the ADHD group and one in the control) were excluded as their head motion exceeded the motion threshold for this study (described below). All ADHD volunteers were diagnosed with the disorder after an interview performed before initiating imaging. For a patient to be diagnosed with ADHD, he/she should fulfil the diagnostic criteria of the Diagnostic and Statistical Manual of Mental Disorders, Fifth Edition, (DSM-5). This was so for all the symptoms of inattentiveness, hyperactivity and impulsivity. On top of these requirements symptoms for ADHD infants were required to be present practically on each day of the week for the last six months. Also symptoms must have been inducing a dysfunction of the day to day normal activities. Finally, no other diagnosis should explain the symptoms. Once this was established in an interview two tests were always performed on patients: Conneŕs and ADHD rating scales (ADHD-RS). In some occasions the Wechsler (WEISC, to indicate IQ in normal levels) and the TOVA were also applied (data not presented here). All ADHD volunteers had never received medication for this disorder. Only volunteers with ADHD of the Inattentive and the Impulsivity-Hyperactivity subtypes were considered for this study. Controls were completely healthy at the time of the study, with no history of any previous psychological or mental disorder and no prior neurological interventions. No intelligence matching between groups was performed.

The 46 infants finally involved in this study had an average age of 9.7±2.4 (6–12 years old), (mean ± standard deviation, (age range)), with the ADHD group (n = 23) being 9.3±2.8 (6–12 years old), and the H group (n = 23) 9.3±3.5 (6–11 years old) on average. There was a predominance of male subjects, as just 4 out of 46 volunteers were female (2 in the ADHD group and 2 in the H). ADHD patients of the inattentive subtype scored in the ADHD-RS scales 38.0±3.1 and in Conneŕs 66.1±2.8. ADHD patients of the mixed subtype scored in the ADHD-RS scales 38.3±4.0 and in Conneŕs 69.4±1.3. All patients were selected randomly from the population which checked in the Neurology department of the Hospital Infantil de México, Federico Gómez (Mexico). Due to the nature of the hospital being the public national reference in children care; volunteers belonged to all the social, economic and cultural backgrounds found in Mexican society. Nevertheless, no test or scale was used to support this fact.

### Hardware

Experiments were performed on a 1.5 T Philips Intera-Achieva scanner (Philips, Inc., Netherlands), using an 8 channel SENSE head-coil. Gradient coils were NOVA (Copley 271 Dual, slew rate 80 mT/m/ms, peak amplitude 120 mT/m).

Protocol: Resting state scans were acquired immediately after standard setup of the scanner and patient, with the volunteer in a supine position with closed eyes (but always awake). The resting state sequence was the first one to be run on all patients and lasted for a period of 7 minutes 25 seconds. Following this sequence a fast anatomical image was acquired which lasted for 3 minutes 10 seconds. Once these sequences were acquired patients were extracted from the scanner.

### MRI Sequences

Resting State: 150 whole brain volumes, comprising 35 axial slices covering the whole of the brain (including the cerebellum), were acquired with a fast-echo EPI sequence. TR = 2.9 s, TE = 50 ms, 90 degree flip angle, 64×64 matrix with a 3.6×3.6 mm *in-plane* resolution and 4 mm slice thickness (no gap between slices). Anatomical images were acquired with a T_1_-weighted gradient echo sequence (TR = 307.81 ms, TE = 2.48 ms, 4 repetitions and 80 degree flip angle). Sequences covered the same FOV with a 640×640 matrix which gave a 0.36×0.36 mm *in-plane* resolution and 4 mm slice thickness (also no gap between slices).

### Data Analysis

Resting state network data analysis was performed with: Data Processing Assistant for Resting State fMRI Advanced edition (DPARSFA_V2.2; [39]; http://www.restfmri.net), Resting-State fMRI Data Analysis Toolkit (REST1.8; [40]; http://www.restfmri.net) and Group ICA of fMRI Toolbox (GIFT; [23]; http://mialab.mrn.org/index.html). All software programs were run on a Statistical Parametric Mapping platform (SPM8; http://www.fil.ion.ucl.ac.uk/spm), based on the Matlab programming language (The Mathworks Inc., USA).

### Image Analysis

Resting state network analysis began by converting all DICOM files obtained from the scanner into the ANALYZE format. In the process the first 7 volumes of functional data were discarded to avoid signal saturation problems (each subject had 143 useful sets of volumes). Slice timing correction followed on the 35 slices (ascending order, centered on the middle slice). It has been shown previously that spurious systematic correlations arise due to head motion during experimentation [41]. The DPARSFA realignment step was based on the correction used by SPM8 (a standard least squares approach using a rigid body approximation with six motion parameters). To this correction, a voxel-specific head motion calculation was added which performed frame-wise displacement measurements (FD) of head motion as introduced by Power et al. [42]. This was done at volume and at voxel levels (always relative to the previous time point). It has been demonstrated that when FD values of volumes were over 0.5, artifactual BOLD correlations appeared in the results. Therefore a threshold for head motion during this study was established at 3.5 mm and/or 3 degrees shift during the experiment together with the requirement that no volume in a subject was allowed to have an FD value over 0.5. To these thresholds of motion a data scrubbing step executed before image analysis (before Alff and ReHo) was performed to reduce even more significantly motion effects. In it, all volumes with FD voxel values over 0.5 were deleted from the analysis together with the previous and consecutive volumes. Finally a parallel study to this image analysis performed with the artifact detection tool (art, www.nitrc.org) showed that the number of volumes that should be eliminated due to motion at voxel level was 5.73±6.15 (mean ± standard deviation) for the control group and 9.32±8.25 for the ADHD group. As suspected the ADHD group had more movement than their control counterparts. Nevertheless this difference did not reach statistically significance (p = −0.146, t-test) limiting the effect that motion could have on results for this study. Motion values of each individual during scanning were incorporated to the analysis further on, in the regression of nuisance covariates step. Anatomical and functional images of each volunteer were co-registered using interpolation and DARTEL methods [43]. In this step the anterior commissure was considered as the reference point in the brain. The quality of the co-registration was assessed visually by a researcher in each case by overlaying anatomical and functional data in a display window for each volunteer. All the anatomical data was then combined together and used to segment white matter, grey matter and CSF. The CSF and white matter segmentation data were applied as masks to the functional data of each volunteer in a regression of nuisance covariates step, which eliminated these sources of noise. The use of global signal correction has been controversial in the field [44] and was not used in this analysis. Nuisance regression was done prior to filtering for low frequencies, to avoid the creation of artifacts due to these random sources remaining in the data. During this step, data was also detrended for linear and quadratic drifts. With the denoised data, DPARSFA software was used to wrap the masks of each individual into their own space. An ALFF (amplitude of low frequency fluctuations; [45]) calculation of the BOLD fluctuations between 0.01 and 0.1 Hz was then performed on each volunteer. These analyses gave an idea of the activity of different regions in the resting state network results. Regions of homogeneity (ReHo) or areas of correlated voxels were calculated using up to 27 neighbors for each voxel and employing the ReHo analysis toolkit of DPARSF (ReHo analysis; [12]). Once all of the functional analysis was completed for each volunteer, all individual data, masks and results were normalized to a standard Montreal Neurological Institute (MNI) brain template using the DARTEL approach. Finally, in order to reduce noise and compensate for anatomical differences when performing inter-subject averages all normalized data was smoothed with a [4,4,4] mm FWHM Gaussian kernel. Image presentation used the viewer option of the REST software application projecting data on the Open Ch2 brain template (also in MNI coordinates).

For ICA analysis, data was first preprocessed. All the data (H and ADHD groups) were analyzed together in these first steps. Slice time and motion correction of data was performed as in the resting state network analysis and was then normalized (estimated and written) to an EPI.nii template in MNI coordinates using SPM8 software. Normalization was performed with a tri-linear interpolation, a bounding box of -78-112-50 mm and voxel sizes of (2, 2, 2) mm. After this, data was filtered to retain frequencies between 0.01 and 0.1 Hz, and smoothed using a (4, 4, 4) mm kernel using DPARSFA software. To complete preprocessing, data was visually assessed using the “Check Registration” option in SPM8, and was fed to the GIFT software. At this point data was divided into three groups; one with ADHD subjects (description of ADHD with ICA), the other with the H volunteers (ICA results from the H group) and a mixed group (all data together to perform second-level statistics of the ICA results between groups). ICA analysis was performed for each of the three subgroups with the following parameters: regular analysis (analysis run only once) with the Infomax algorithm and with 40 components. The number of components selected for ICA analysis is always controversial. This value was selected based on the work of Damoiseaux et al. which found 12 components in healthy patients. This would give our analysis 28 components more to show the possible appearance of new networks. All the analysis steps that this software follows were completed (parameter initialization, group reduction, ICA calculation, back reconstruction, component calibration and group statistics). Finally each individual component (average of the same component for all the subjects) was displayed on top of the EPI.nii template image. A threshold of Z>3.33 was used to eliminate random results. All 40 components were visually assessed. 12 were regularly found in all groups and considered to be significant as they corresponded to networks reported by Damoiseaux et al. The other 28 components were considered to be noise (correlations in structures outside of the brain) or motion (correlations on the borders of brain structures like in brain ventricle walls or in the limit of imaged volumes), and were discarded.

Second-level analysis was used to compare the different components between the ADHD and H group. It was implemented using the statistical capabilities of SPM8, which were accessed through the utilities option in the GIFT software. Here, two sample Mann–Whitney U tests were performed between the same components of the ADHD and H mixed group. This way, component 9, for example, was the same for both the H and ADHD groups. Results of the comparison for each component were also thresholded at Z>3.33 and projected onto the EPI.nii template. For publication purposes, each significant component was displayed using the “View” option of the REST software (5×5 images with 7 mm separation between slices that covered the whole brain including cerebellum).

### Statistics

All data studied failed normality tests; therefore non-parametric statistical tests were applied. When comparing voxel numbers and images, Mann–Whitney U tests were used with a threshold of p<0.001 (Z>3.33) corrected for multiple comparisons. For display purposes images were subject to a p<0.01 (Z>2.58) threshold (also corrected for multiple comparisons), with the added requirement of at least 4 responding voxels lying together to consider the activation as a positive result [46]. The requirement of 4 voxels lying together can increase five-fold the statistical power of a result in some cases [47]. Multiple comparison corrections were performed with family wise error methods (FWE) available in SPM8 and GIFT. The p threshold value selected was similar or stricter than those used in other publications in this field (i.e. [12], [30]).

## Results

**Figure 1:**
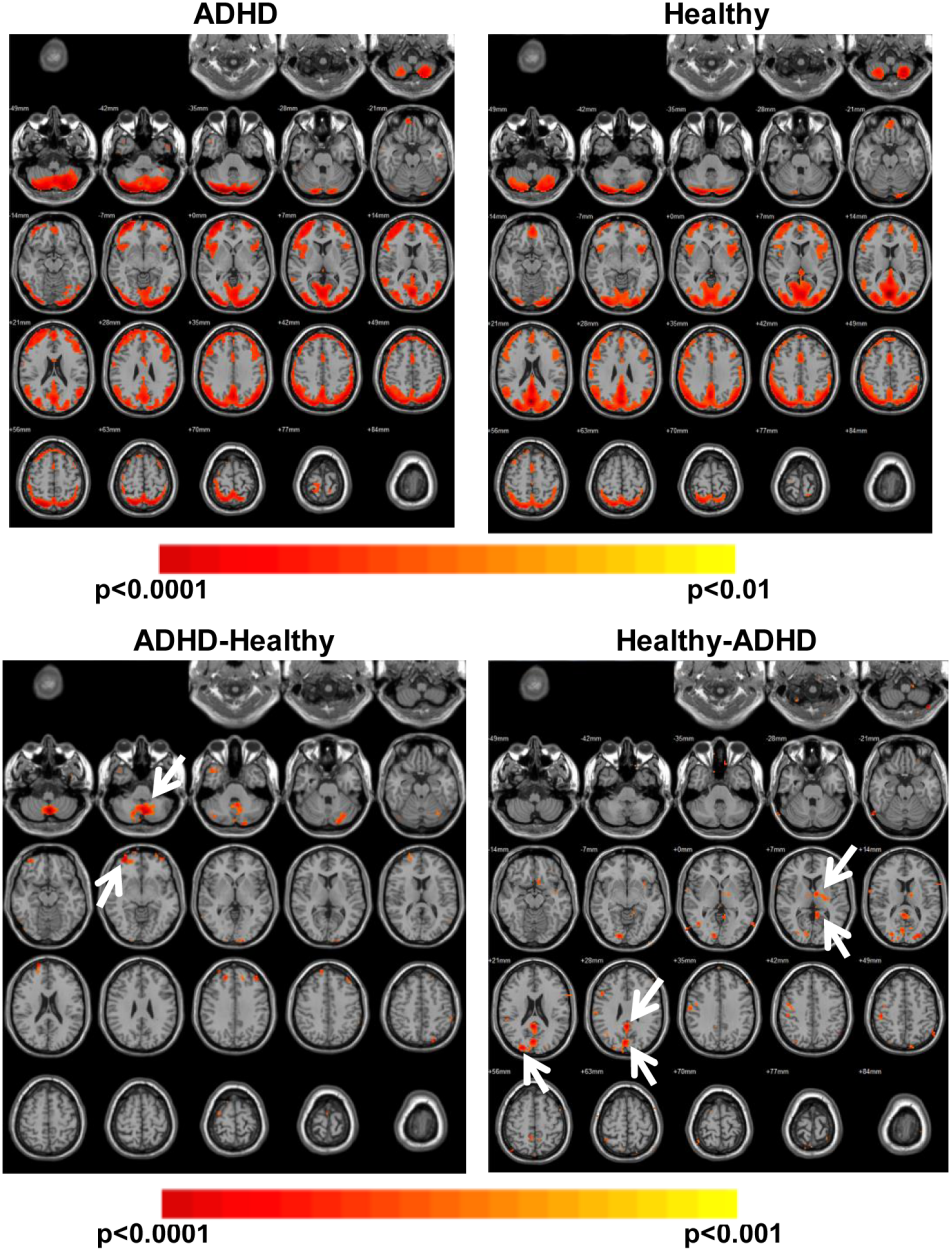
Region of Homogeneity (ReHo). ReHo results are presented in this image. The first row shows averaged data for the ADHD and H groups. Pseudo-colored images (red color scale) are threshold at p<0.01 (corrected for multiple comparisons). The second row presents Mann–Whitney U test comparisons between the images of the two groups, ADHD-H and H-ADHD. These images are threshold at p<0.001 (corrected for multiple comparisons). They show relevant regions of difference between the groups, with the most significant ones highlighted by white arrows.

**Figure 2:**
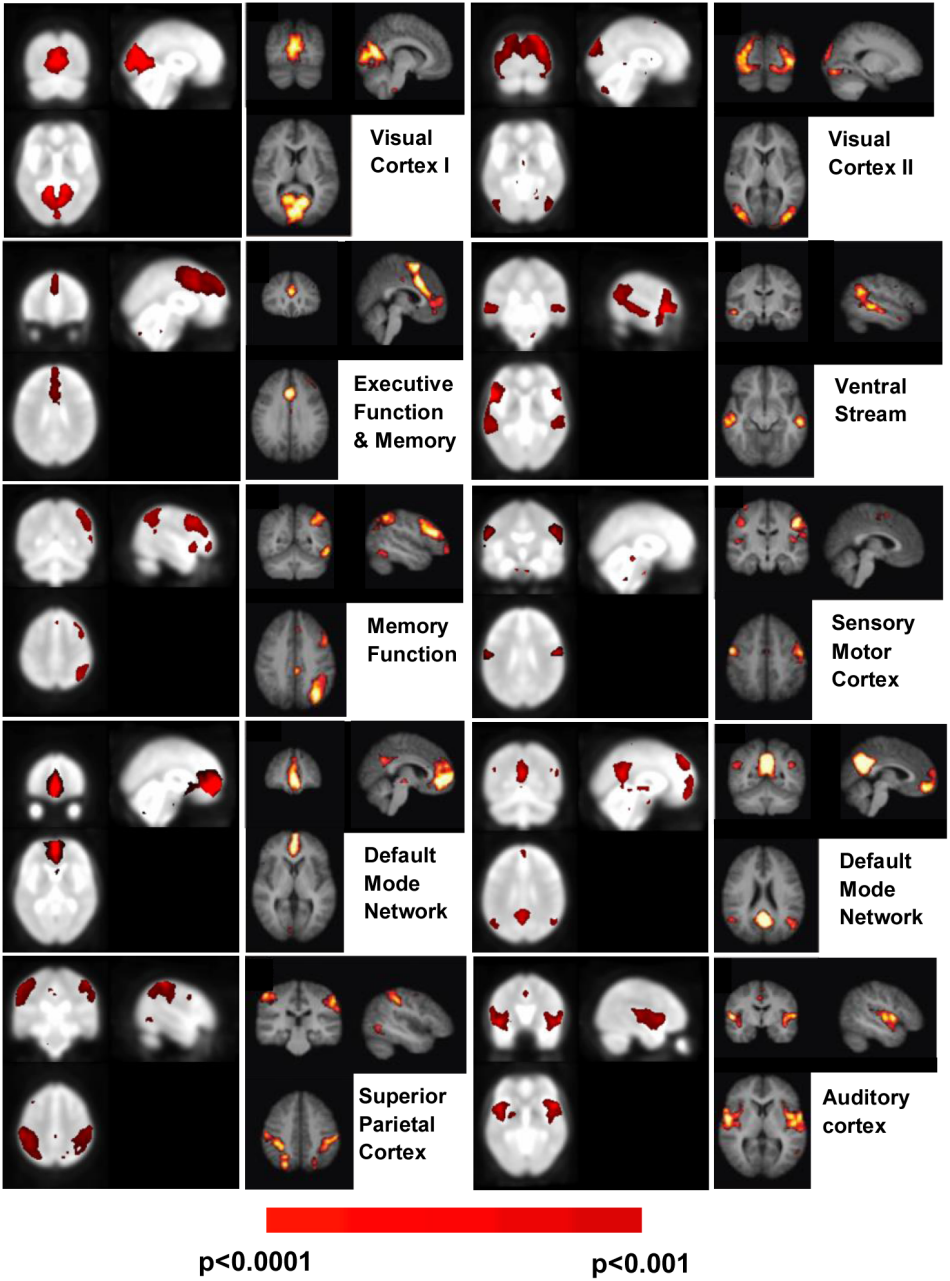
A comparison of ICA results between healthy adult subjects and the group of ADHD children. This figure presents the ten principal ICA networks found in healthy adult humans, as presented by Damoiseaux et al. It compares them with the results from our ICA analysis of the ADHD group. On the left of each panel the results for the ADHD group can be seen and compared with the right panel, where the network obtained by Damoiseaux appears with its corresponding name. Results were thresholded for the ADHD group at Z>3.33, corresponding to p<0.001. Only the Right Memory Function network is presented for simplicity, but a similar Left Memory Function network exists.

The first objective of this study was to perform a comparison between resting state networks of ADHD patients and age matched healthy controls using the ReHo methodology. Results (Figure 1, top row) showed similar networks for both groups. Values of Z score varied between 2.58 (p = 0.01) and 4 (p<0.0001) in both cases. In the first row of this image the average image for ADHD patients showed a larger number of total voxels correlated when compared to the H group. This difference was 26% at the thresholds used. In order to quantify this effect, data from each subject of the two groups was studied. No statistically significant difference was found between the two groups in this analysis. Significant areas in both cases were: the occipital and frontal cortex almost completely, the inferior and medial temporal cortex, anterior and posterior cingulate cortex, as well as the cerebellum, cuneus, precuneus, motor cortex and insula. All the regions listed above presented p<0.001 after correction for multiple comparisons. In Figure 1 bottom row, results from a comparison between the two groups showed the presence of regions with significantly different correlation values. These values were greater for the H compared with the ADHD subjects in some regions (precuneus, cuneus, left mid-occipital cortex, right putamen, left lingual and ventral pallidum), and lower than ADHD subjects in others (cerebellum lobes 8 and 9 as well as the cerebral crus and the medial frontal cortex bilaterally). All the regions listed above were obtained as a result of a Mann–Whitney U test comparison, with p values under 0.001 after correction for multiple comparisons. In Figure 2, ICA networks obtained from the ADHD group of this study were compared with the resting state networks of healthy volunteers presented by Damoiseaux et al. [8]; ten healthy volunteers with ages of 22.8±2.3 years old). Values of the Z score varied between 2.58 (p = 0.01) and 4 (p<0.0001). All the networks that were present in the Damoiseaux article appeared in this study. The Executive Function & Memory, Ventral Stream and Visual Cortex II networks did not show differences with respect to the control networks and could be clearly recognized. The Ventral Stream network showed new bilateral activations in the ADHD group, in a region that corresponded to frontal inferior orbital cortex. The Executive Function network in this study took up parts of the frontal cortex and did not reach the medial parts of it. ADHD’s Visual Cortex II networks invaded medial structures in the occipital/parietal lobe. Finally, the default mode network was found here to have three components and not just two (Figure S1). In this image, calculated and presented in the same manner as Figure 2, there was a third component for the Default Mode network (named Default 3) which covered the cuneus and precuneus regions as well as the angular and superior parietal cortex (bilaterally).

**Figure 3:**
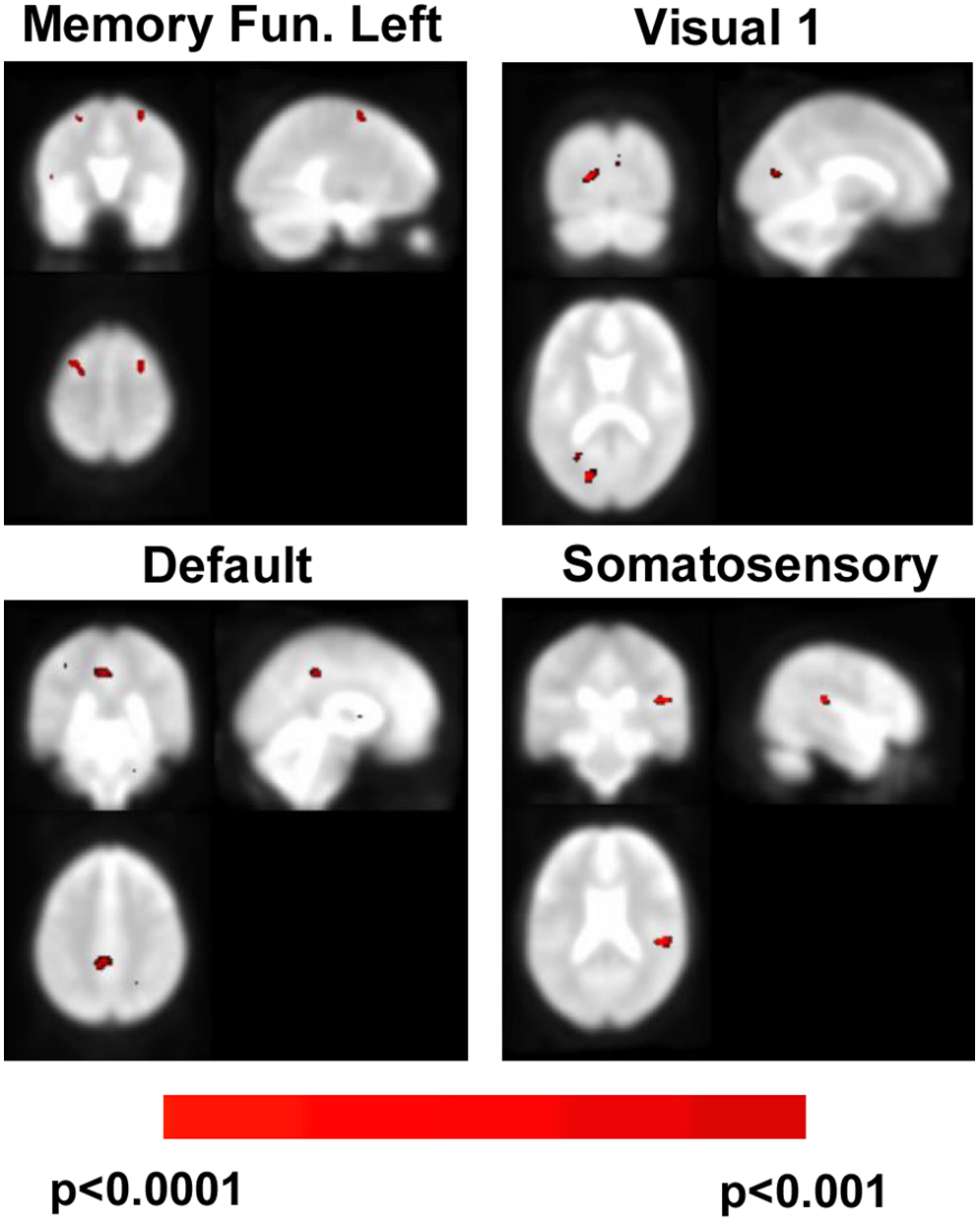
Second-level analysis comparing the ICA results of the H and ADHD groups. This figure presents four sample regions that were found to be statistically significant (p<0.0001, U-Mann Whitney tests) in the ICA analysis of the H group over the ADHD group. These regions are the left temporal superior lobe (72, 49,45, MNI coordinates) in the Somatosensory network, the right calcarine fissure (33, 33, 43) in the Principal Visual network, the mid-frontal lobe (33, 68, 68 and 54, 68 68) for the Left Memory Function network and the right middle cingulum (45, 45, 58) for the Default network.

In order to assess the differences between the ICA studies of the ADHD and H groups, Mann–Whitney U tests comparing the different networks were performed. As before, p values were thresholded at 0.001 after correction for multiple comparisons. In Figure 3, examples of significant brain ROIs recruited differently for each group can be observed (i.e. Visual 1, Default, Somatosensory and Left Memory Function networks).

A list with the position in MNI coordinates, number of significant voxels as well as the names that correspond to these regions can be seen in Table 1. All the networks showed significant *inter-group* differences, except for the Right Memory Function network. In general the majority of these differences between networks were found in the frontal and temporal cortices, as well as cuneus, precuneus and the lingual regions.

**Table 1:** Second-level analysis comparing the ICA results of the H and ADHD groups.

Network	ADHD>H	H>ADHD	Number of Voxels	Regions Name
**Auditory**	48, 85, 59		88	Superior medial Frontal R
		19, 71, 36	23	Superior Temporal R
**Somat. Cortex**	27, 86, 41		12	Inferior Frontal Tri R
	69, 56, 34		17	Mid-Temporal L
	40, 35, 64		32	Precuneus R
		72, 49, 45	62	Temporal Superior L
**Executive Function**	58, 30, 35		25	Lingual L
	47, 96, 46		22	Mid-Superior Frontal
	71, 54, 43		28	Temp Superior L
	42, 39, 74		19	Paracentral Lobule R
		71, 60, 37	40	Temp Superior L
		26, 76, 60	23	Mid-Frontal R
		50, 34, 39	15	Lingual L
**Visual 1**		40, 25, 44	102	Calcarine R
		74, 59, 60	15	Precentral L
		33, 33, 43	21	Calcarine R
		54, 27, 56	19	Cuneus L
**Visual 2**		35, 14, 42	22	Mid-Occipital R
**Memory Fun. Left**	77, 47, 38		17	Superior Temporal L
	27, 46, 59		14	Postcentral R
	60, 89, 56		19	Mid-Frontal L
		33, 68, 68	16	Mid-Frontal R
		62, 68, 68	16	Mid-Frontal L
		18, 77, 40	30	Inferior Frontal Tri R
**Ventral Stream**		72, 63, 66	46	Precentral L
		63, 73, 39	20	Insula L
		54, 60, 75	15	Precentral L
**Sup. Parietal Cortex**	23, 73, 58		25	Inferior Frontal Operculum R
		34, 45, 23	20	Cerebellum 4_5_R
**Default 1**	41, 95, 41		17	Mid-Superior-Frontal L
		71, 49, 61	77	Inferior Parietal L
		23, 30, 59	20	Angular R
		67, 93, 37	19	Mid-Orbito-Frontal L
		40, 97, 48	15	Mid-Frontal R
**Default 2**	69, 37, 63		34	Parietal Inferior L
	25, 36, 19		17	Cerebellum Crus 1_R
	45, 23, 58		13	Cuneus R
		55, 38, 13	22	Cerebellum_8_L
**Default 3**	63, 42, 31		18	Fusiform_L
	51, 82, 62		21	Frontal Superior Medial L
	18, 74, 49		30	Frontal Inferior Tri R
		45, 45, 58	90	Mid-cingulum R
		61, 32, 36	52	Lingual L
**Memory Fun. Right**	NONE	NONE		

This table presents the different regions found when performing a comparison between the ICA results of H and ADHD groups. After comparison (p<0.0001, Mann–Whitney U tests), the resulting regions are presented with the following information: ICA network name, ADHD>H or ADHD<H, position of maxima in MNI coordinates, voxel number, and finally region name.

**Figure 4:**
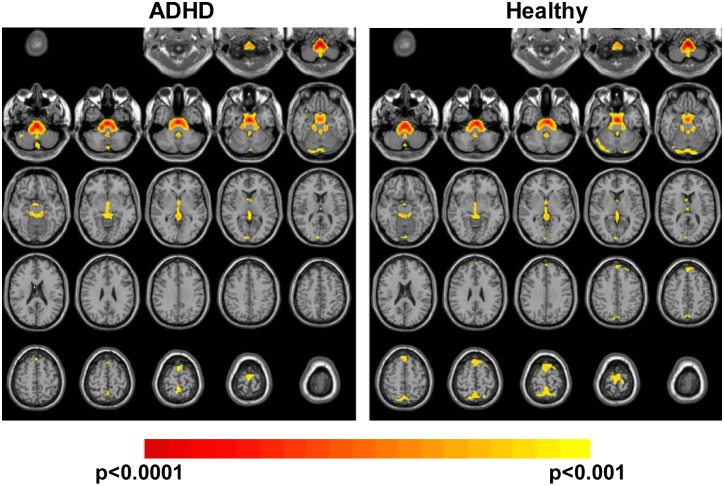
Amplitude at low frequency (ALFF). Here ALFF results with pseudo-colored images (red color scale) are presented. The first row shows averaged data for the ADHD and H groups. Images are threshold to Z>2.58 (p<0.001 corrected for multiple comparisons).

In Figure 4 results from the ALFF study comparing, once again, the H and the ADHD group are presented. The difference between this analysis and those presented previously is that it can be considered as a measurement of function or activity of the different brain regions and not a correlation. In the only row of this figure the average results for both groups can be found. Data was thresholded to a Z value between 2.58 (p = 0.01) and 4 (p<0.0001) in both cases. Significant amplitudes could be found in both cases in deep brain structures such as the pons and the medulla oblongata. Significant amplitudes were also found in both cases in the cerebellum (cerebellar crus 2 and vermis 1) together with the right calcarine fissure and the precuneus. All the regions listed above were significant at p<0.001 corrected. No differences with respect to areas and magnitude of amplitudes between the two groups were found (Mann–Whitney U test for comparison of images, data not presented).

## Discussion

In this project the resting state networks of pre-treatment ADHD children (aged between 6 and 12 years old) and sex-matched controls were compared. The objective of this work was to highlight some of the physiological differences of the ADHD brain when compared to a healthy one. Different image analysis techniques were used for this study (ReHo, ICA and ALFF). The following major differences were found: ReHo and ICA analyses identified differences between the two groups while ALFF analysis did not. ReHo and ALFF analysis showed different regional recruitment in the ADHD group of this study when compared to previous work. ICA analysis showed that the same resting state networks that appear in healthy volunteers of adult age were obtained for both groups in this study. These networks were not identical and included changes such as the appearance of a third component of the Default network. When comparing healthy and ADHD groups, ICA analysis also showed differences in responding areas for all the networks except for the Right Memory Function network.

### Differences between H and ADHD Groups Obtained with ReHo Analysis

When considering ReHo activations, which were greater for the H group compared to the ADHD patients, the following regions were statistically highlighted: cuneus, precuneus, occipital cortex, right putamen and right globus pallidus. The relevance of these findings is consistent with the difficulty that ADHD patients have to process visual and spatial inputs (precuneus), as well as to plan and organise movements (putamen & cerebellum). Also, the precuneus and cuneus are important nodes of the default mode network. The precuneus has been previously reported to have reduced connectivity for ADHD patients when compared to controls [48]. The cuneus is also affected by ADHD [49]. The results in our study corroborate all of these findings.

The putamen and globus pallidus, which form the lentiform nucleus with their high level of connectivity are involved in several functions such as reinforcement, learning, concept learning, motor function, etc. The globus pallidus controls, through its inhibitory function, the positive activity of the cerebellum in movement. It is also known that ADHD affects it as well as the putamen [50]. Probably this de-regulation of the inhibitory function of the lentiform nucleus is what is being observed in these results; thereby presenting a positive correlation in the globus pallidus of the healthy subjects which is not observed in ADHD patients. Recently the putamen has been involved in a new, specific ADHD resting state network involved in information integration and emotional control [51]. The globus pallidus, in contrast, has been known to present different degrees of centricity (number of connected voxels; see [52] but was never reported to have a different ReHo.

When considering which ReHo activations were greater for the ADHD patients when compared to the H, the following regions were statistically highlighted: cerebellum (crus and lobes 8 and 9) and mid-frontal cortex, bilaterally. The cerebellum is known to be involved in planning, organization and execution. It is also known to control the calibration of motor function and learning by inference. Both of these last two functions are altered in ADHD patients. The excess of coherence found in the cerebellum for the ADHD group indicates a neurological basis to this effect. This “dysfunction” of the cerebellum is accompanied by a reduction in its volume [53], especially in areas in which this study found large correlations for the ADHD group (lobes 8 to 10).

It is important to notice that ReHo correlations did appear in both groups in the same areas. Differences were driven exclusively by the strength or magnitude of the correlations, and not because of the lack or absence of a correlation in a given region.

### Differences between Healthy Adult Volunteers and ADHD Patients Obtained with ICA Analysis

No new networks were found for the ICA analysis of ADHD patients when compared to healthy adult volunteers (Figure 2). Therefore no new resting state networks associated with the underlying neurobiological mechanisms of ADHD were found in this experiment. Nevertheless, almost all networks showed significant changes between groups.

The Visual Cortex II network invaded medial structures of the occipital lobe that corresponded mainly to parietal cortex and its function as a convergence point between vision and proprioception. It also showed lower correlations in areas like Brodmann 18 and 19, which are secondary and tertiary regions related to image association. The Ventral Stream network showed new bilateral activations in the ADHD group in a region that corresponded to the frontal inferior orbital cortex (Brodmann area 47). Both of these findings supported previous studies in which ADHD children showed impaired early visual processing [54].

The Executive Function network of ADHD patients took up areas of the frontal cortex but did not reach the medial parts of this lobe as it did for healthy subjects. This region, which did not show up in the ICA analysis for ADHD patients, would correspond to Brodmann area 10, or equivalently, the frontopolar prefrontal cortex. This is a meaningful result as this region is known to be involved specifically in executive function (as well as strategic processes and memory recall). The lack of connectivity to this region may account for the problems ADHD patients have in planning or using working memory, or with their task flexibility.

A new component for the Default network (Default 3) appeared in both healthy and ADHD groups. The regions involved (angular, cuneus, precuneus and superior parietal lobe) were areas which appeared in the two previous default networks found by Damoiseaux et al. Based on these two considerations it could be argued that this third component is just a manifestation of the different results obtained when using different image analysis methodologies (i.e. it is a mathematical and not a biological result).

It is nevertheless of interest to highlight the difference in connectivity of the medial cingulate cortex, which in H was larger than in ADHD patients. It is known that damage to this region can induce mental disorders as well as affect information integration (cognitive and affective disorders), and our findings corroborate this.

### Differences between H and ADHD Groups Obtained with ICA Analysis

Differences between the networks of the two groups were broad and statistically significant (with the exception of the right memory function network).

The visual networks (Vision I and II as well as the Ventral Stream) included eight regions which were involved in image processing in healthy subjects that did not appear for the ADHD patients. The most consistently observed regions amongst these eight were the calcarine sulcus, the precentral gyrus and areas in the occipital lobe. The calcarine sulcus is where primary visual cortex is located in the brain [55], and the occipital lobe is an association area for vision. These findings highlight the problems that ADHD patients show with visual information integration.

Lenz et al. [54] found that image processing inability for ADHD patients was associated with the lack of an early memory classification of problems. In the ICA analysis of this study, no changes were found in the Right Memory Function network between groups, but gross changes were found for the left network. From this, we might conclude that healthy subjects made primary use of the frontal lobe, while in ADHD patients a mixture of regions were involved, including the frontal and temporal lobes. In a study by Spinelli and colleagues [56], auditory cortex as well as the precentral gyrus were recruited by ADHD patients when solving problems before the appropriate regions for this task were involved. This could easily explain why the ICA activation on precentral gyrus appeared. We therefore argue that image processing is impaired in ADHD patients due to three factors. First, the lack of inputs from V1 as well as the malfunction of some association cortex areas. Second, the recruitment of regions which are not related to image processing, such as precentral gyrus and Brodmann 47 (as seen in Figure 2). Finally, an inappropriate mixture of recruited areas for memory tasks that exclude the frontal areas of the cortex.

Executive function is related to problem solving, planning, working memory, reasoning and task flexibility. When comparing controls to ADHD patients, the latter presented in ICA analysis a strong correlation in what would correspond to the paracentral lobule. This is a meaningful result as this region is known to have reduced connectivity in ADHD patients [27]. This result, combined with the fact that all the other statistically significant regions (lingual, frontal and temporal lobes, see Table 1) appeared in both groups, indicates that ADHD patients were unable to recruit this region for executive function processing.

### Differences between H and ADHD Groups Obtained with ALFF Analysis

In this study no significant differences in ALFF activity was found between the two groups. This indicates that for the population of this study, ADHD did not affect the function of the different regions of the brain. This is in contrast with previous work comparing non-medicated ADHD patients to healthy ones, which showed larger activations for healthy in sensory motor cortex, frontal cortex and cerebellum [30]. It also showed larger activations for ADHD in brainstem, anterior cingulate cortex and cerebellum.

ALFF analysis did show that regions in the brainstem (pons and medulla oblongata), which had previously been mentioned in literature for ADHD patients, were also active here. In contrast, a group of regions not previously reported were found to be active for both groups. These were the precuneus, cuneus and the calcarine fissure. Except for the calcarine fissure, all the other regions were also found in the ReHo analysis and were found in both groups at a similar magnitude. These results could be an indication that ADHD does not affect drastically the function of a region. It could be argued that it is the inter-regional connectivity, as well as the areas recruited for a given function, which are altered by the disorder.

### Differences with Respect to Previous Work

We believe that the main differences found between this study and previous work arise mainly because of the population used, and methodological issues.

Even if the patient sample used here is in general larger than those used in the past, there are plenty of biological factors that might affect the results and produce differences, such as age, medication, subtype of ADHD, IQ, emotional situation of the patient, genetic origin, etc. As mentioned before, patients in this study had ages between 6 and 12, while the majority of previous studies were performed on patients with ages between 11 and 18 years old. There is a large difference in brain physiology of children and adolescents [57], and some of the differences highlighted as different findings of the ReHo analysis could have this origin. The different subtype of ADHD studied is also a factor to be considered. To our knowledge all work that has been previously performed has considered ADHD patients as a homogeneous group without any subdivision into one of the three subtypes. Considering that ADHD patients from just two subgroups were used here, this could certainly account for some of the differences.

The methodological issues should also be considered (ICA vs. ReHo, ICA vs. ALFF, kind of platform used for analysis, etc.). These are complicated computational analyses in which several (if not hundreds) of parameters have to be set by the researchers. It is impossible for the methods section of an article to present all of this information. Therefore differences between studies will appear due to this factor.

## Conclusions

In general, results from the three techniques indicated that the cerebellum and mid-frontal lobe bilaterally for ReHo, the executive function regions in ICA and the precuneus, cuneus and the clacarine fissure for ALFF, were the “hubs” through which ADHD affected brain function. Results from ReHo also showed that regions like the cuneus, precuneus, occipital cortex, right putamen and right globus pallidus were significantly important. In fact, ReHo correlations were always present for both groups in all regions and just presented different strengths, indicating different connectivity but not a complete malfunction of the region in question. ALFF results indicated that in general, function in every region was the same when comparing healthy controls and ADHD patients.

Based on our results, the techniques used in this study could be considered part of the spectrum of techniques employed in the assessment of attentional disorders. The combination of them has shown that they are complementary and provide different views of the problem. This opens a door to more objective studies for ADHD diagnosis and the understanding of its physiopathology. In the near future the methodology used here could represent a helpful diagnostic tool to monitor the neurobiological changes of this disorder over time and during drug treatments. It was of great interest to observe how connectivity and dysfunction of several brain regions changed. This supported a context in which ADHD was a neurodevelopment disorder, and shows how early-stage intervention could help balance these intricate compensatory neuronal networks.

## Supporting Information

Figure S1 **Default network components obtained from the ICA analysis of this study.** In this image the three components into which the Default network was found to be divided in this study are presented. The first two are compared to the components obtained from Damoiseaux et al. (right panels), while the third stands alone. Data was thresholded between p<0.001 and p<0.0001, and a pseudo-colored bar indicates these differences.
(TIF)
