# Correction: A Multi-Methodological MR Resting State Network Analysis to Assess the Changes in Brain Physiology of Children with ADHD

**DOI:** 10.1371/journal.pone.0143785

**Published:** 2015-11-23

**Authors:** Benito de Celis Alonso, Silvia Hidalgo Tobón, Pilar Dies Suarez, Julio García Flores, Benito de Celis Carrillo, Eduardo Barragán Pérez

There is an error in Table 1 The values for position of activations for columns ADHD>H and H>ADHD have been corrected. Please view the correct Table 1 here.

**Table:** 

Network	ADHD>H	H>ADHD	Number of Voxels	Regions Name
**Auditory**	4, 42, 44		88	Front Sup Med R
		-54, 14, -2	23	Temp Sup Pole L
**Somat. Cortex**	-38, 44, 8		12	Front Mid L
	46, -16, -6		17	Temp Sup R
	-12, -58, 54		32	Precuneus L
		52, -30, 16	62	Temp Sup R
**Executive Function**	24, -68, -4		25	Lingual R
	2, 64, 18		22	Front Sup Med L
	50, -20, 12		28	Temp Sup R
	-8, -41, 77		19	Paracentral L
		50, -8, 0	40	Temp Sup R
		-40, 24, 46	23	Front Mid L
		8, -60, 4	15	Lingual R
**Visual 1**		-12, -78, 14	102	Calcarine L
		56, -10, 46	15	Precentral R
		-26, -62, 12	21	Calcarine L
		16, -74, 38	19	Cuneus R
**Visual 2**		-22, -100, 10	22	Occip Mid L
**Memory Fun. Left**	62, -34, 2		17	Temp Mid R
	-38, -32, 45		14	Postcentral L
	28, 50, 38		19	Front Mid R
		-26, 8, 62	16	Front Mid L
		32, 8, 62	16	Front Mid R
		-56, 26, 6	30	Front Inf Tri L
**Ventral Stream**		52, -2, 50	46	Precentral L
		34, 18, 4	20	Insula R
		16, -8, 76	15	Front Supp R
**Sup. Parietal Cortex**	-46, 18, 42		25	Front Mid L
		-24, -38, -28	20	Cerebell 4_5_L
**Default 1**	-10, 62, 8		17	Front Sup Med. L
** **		50, -30, 48	77	Pariet Inf R
** **		-46, -68, 44	20	Angular L
** **		42, 58, 0	19	Front Mid Orb R
** **		-12, 66, 22	15	Front Sup Med L
**Default 2**	46, -54, 52		34	Pariet Inf R
** **	-42, -56, -36		17	Cerebell Crus 1_L
** **	-2, -82, 42		13	Cuneus L
** **		18, -52, -48	22	Cerebell_8_R
**Default 3**	34, -44, -12		18	Fusiform_R
	10, 36, 50		21	Front Sup Med R
	-56, 20, 24		30	Front Inf Tri L
		-2, -38, 42	90	Mid Cingulum L
		26, -61, -2	52	Fusiform R
