# Supplementary figures and images for: A Multi-Methodological MR Resting State Network Analysis to Assess the Changes in Brain Physiology of Children with ADHD
Source: PLoS One. 2014 Jun 19;9(6):e99119. doi: 10.1371/journal.pone.0099119 (PMC4063721; doi:10.1371/journal.pone.0099119)

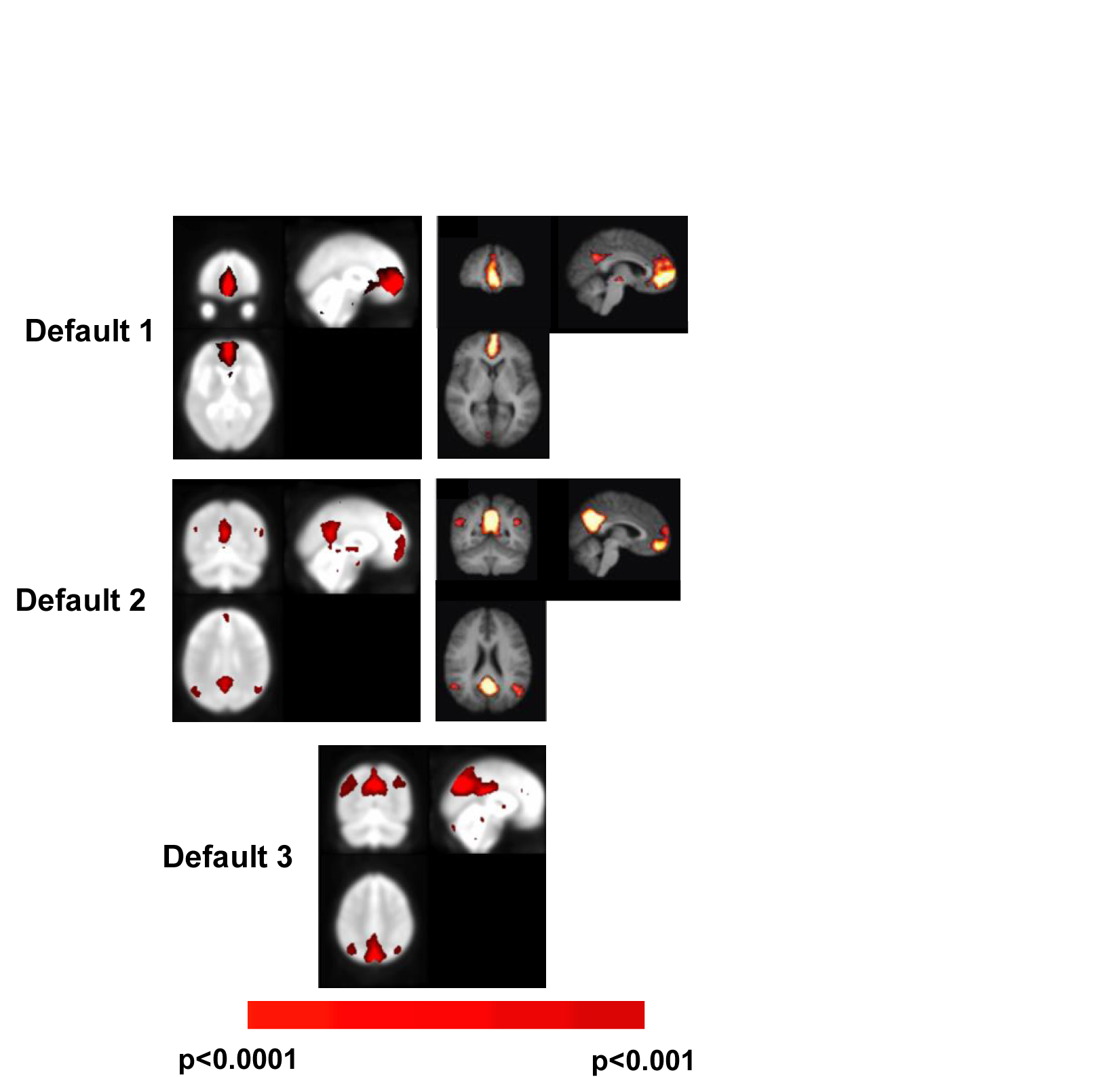

Supplement: Figure S1 — Default network components obtained from the ICA analysis of this study. In this image the three components into which the Default network was found to be divided in this study are presented. The first two are compared to the components obtained from Damoiseaux et al. (right panels), while the third stands alone. Data was thresholded between p<0.001 and p<0.0001, and a pseudo-colored bar indicates these differences. (TIF) [file pone.0099119.s001.tif]
